# Traumatic superior orbital fissure syndrome

**DOI:** 10.3205/oc000099

**Published:** 2019-03-29

**Authors:** Sait Coskun Özcan, Feyza Önder, Nedime Demir, Deniz Özarslan Özcan

**Affiliations:** 1 Mustafa Kemal University, Department of Ophthalmology, Hatay, Turkey; 2 Haseki Education and Research Hospital, Istanbul, Turkey

**Keywords:** superior orbital fissure syndrome, trauma, ophthalmoplegia

## Abstract

**Objective:** Traumatic superior orbital fissure syndrome is a rare complication that occurs in association with craniofacial trauma. In the present case, there were no associated orbital fractures or other lesions to explain.

**Methods:** We present a-16-year-old patient with total ophthalmoplegia, ptosis, and anesthesia of the upper eyelid and forehead 6 hours after a reported trauma to the left eye. We measured the width of the superior orbital fissure on the horizontal plane including the optic canal using computed tomography scans.

**Results:** Radiological examinations did not reveal any orbital fractures. However, the superior orbital fissure on the affected side was only 1.86 mm, increasing susceptibility to indirect trauma.

**Conclusions:** A narrow superior orbital fissure may play a role for superior orbital fissure syndrome altering the transmitted force from the trauma and edema.

## Introduction

The superior orbital fissure syndrome (SOFS) is a complex of impaired function of the cranial nerves (III, IV, V and VI) that enter the orbit through the superior orbital fissure (SOF). SOFS can arise from multiple etiologies and mechanisms. SOFS may be caused by trauma, neoplasia, inflammation, or vascular disorders [1]. Direct bony compression of the contents of the SOF and/or a compression hematoma may cause the signs and symptoms of the syndrome which are either complete or partial depending upon the degree of compression of its related anatomical structure.

We present a case of superior orbital fissure syndrome which is made more interesting by the fact that there were no associated orbital fractures demonstrated on imaging or other lesions to explain. Investigating the cause of the superior orbital fissure syndrome, we found a narrow superior orbital fissure using computed tomography (CT) scans on the affected side. 

## Case description

**Figure 1:**
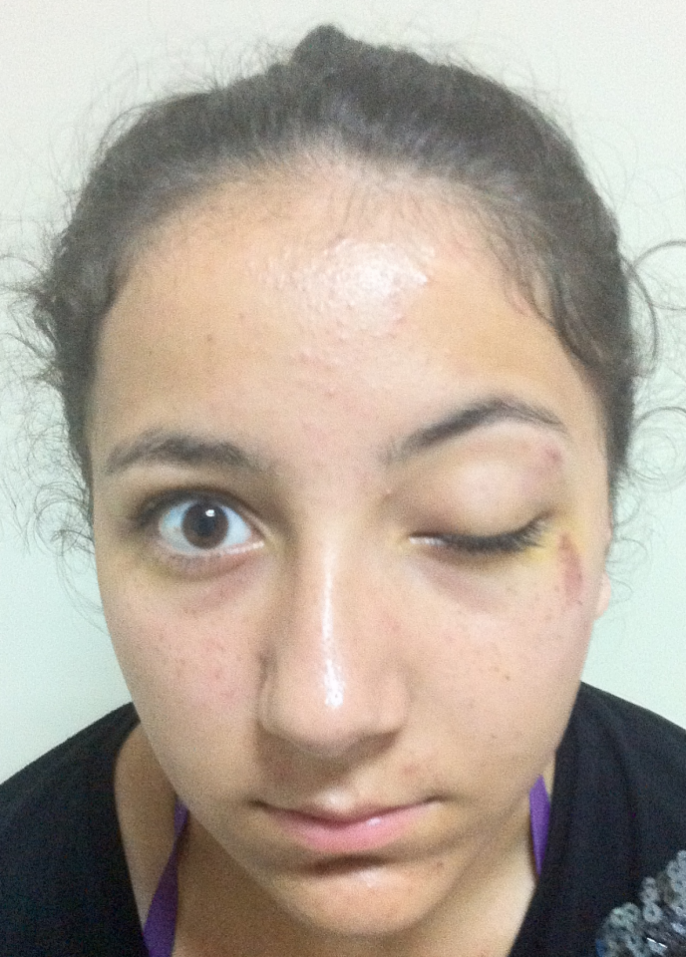
Initial presentation of the patient

**Figure 2:**
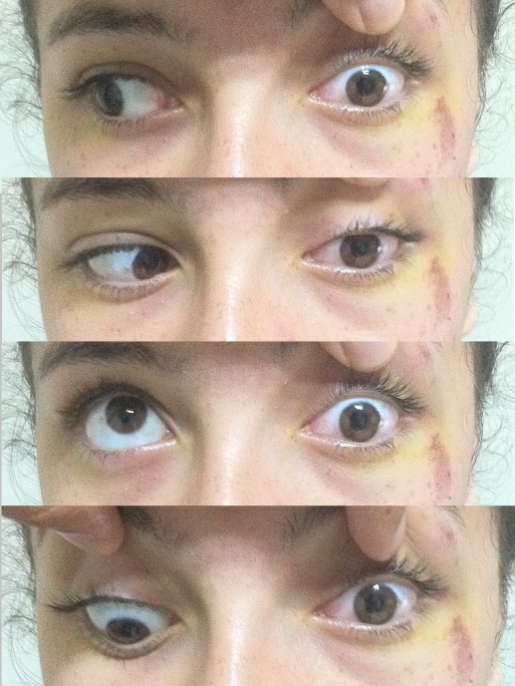
At presentation; total ophthalmoplegia and ptosis of the left eye

A 16-year-old female presented with a complaint of pain, periorbital swelling, and inability to open her left eye. She had suffered injury 6 hours ago. When getting on a bus in traffic, she fell down the bus steps to the floor. She was managed conservatively in a peripheral health center for the head injury and was referred to the ophthalmology unit at our hospital. Examination then showed that she had periorbital edema and complete ptosis on her left eye (Figure 1 (Fig. 1)). Visual acuity was 20/20 in both eyes. There was no proptosis. Color vision was intact. Ocular movements were full in the right eye. There was a complete absence of movement of the eyeball in all the gazes in the left eye (Figure 2 (Fig. 2)). Further ophthalmologic evaluation demonstrated no evidence of optic disc edema, neuropathy, or retinal detachment. She had paresthesia of the left frontal region. She also reported taking no medications and having no known drug allergies or sensitivities.

Clinical examination was otherwise normal. Hematological investigations revealed Hb 12 g/dl and WBC 7,000 cells/mm^3^, chest X-ray was normal, and blood pressure was 110/70 mmHg. The laboratory measures of C-reactive protein (CRP), creatinine and blood urea nitrogen (BUN), blood glucose, lipid studies, B12, folate, thyroid function and other measures were not significant. The erythrocyte sedimentation rate was 4 mm/h.

**Figure 3:**
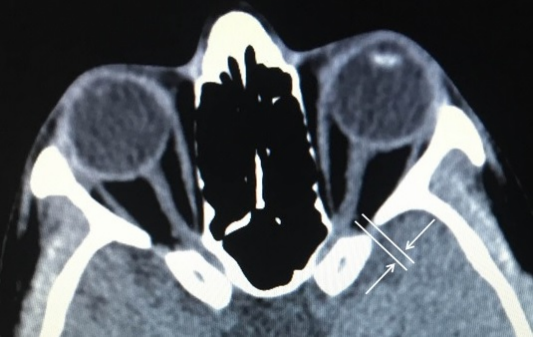
CT scan on the horizontal plane; the arrows indicate the length to measure.

**Figure 4:**
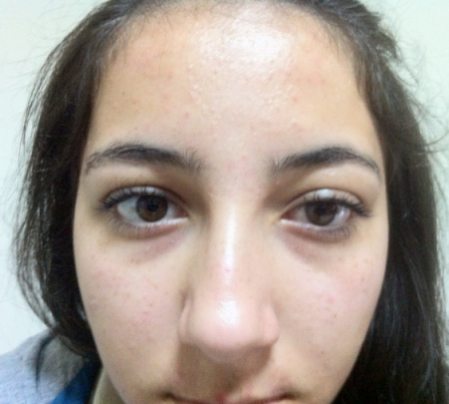
Partial improvement in ptosis and ophthalmoplegia after 2 weeks

**Figure 5:**
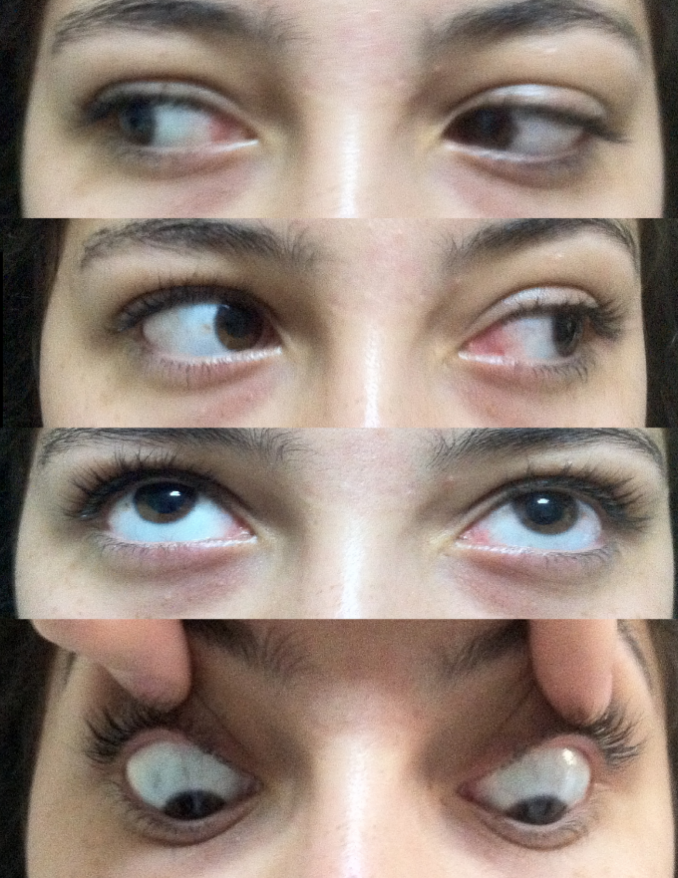
Complete recovery from ptosis and ophthalmoplegia after 10 weeks

The CT scan of the orbits and the maxillofacial area showed no haematoma or fracture of the superior orbital fissure or the surrounding orbital bones. The width of the superior orbital fissure was 3.01 mm on the right side and 1.86 mm on the left side (Figure 3 (Fig. 3)). The diagnosis of traumatic superior orbital fissure syndrome was made and the patient was managed with intravenous 500 mg prednisolone for 3 days. There was a partial improvement in ptosis and ophthalmoplegia after 2 weeks (Figure 4 (Fig. 4)). The patient was followed at regular intervals and her condition improved over the period. At the end of 10 weeks, there was a complete recovery from ptosis and ophthalmoplegia (Figure 5 (Fig. 5)).

## Discussion

The superior orbital fissure (SOF) is bound laterally by the greater wing of the sphenoid, medially by the lesser wing of the sphenoid, and superiorly by the frontal bone. SOF serves as a pathway between the orbit and the middle cranial fossa. The size of the SOF in an adult is around 22 mm in length, 2–3 mm in width at the apex, and 7–8 mm at the base [2]. The tendons of the lateral rectus muscle divide the fissure into two parts: the superior part containing the trochlear, the frontal, and the lacrimal branches of the ophthalmic division of the trigeminal nerve, and the superior orbital vein; and the inferior part containing the superior and inferior branches of the oculomotor, the abducens nerve, the nasociliary nerves, and the inferior orbital vein [3]. 

According to Kurzer and Patel, the superior orbital fissure syndrome was first described by Hirschfeld in 1858 [4]. SOFS is a complex consisting of periorbital swelling, proptosis, ptosis, numbness of the forehead, and paralysis of the extraocular muscles due to impairment of III, IV, VI, and the first division of V cranial nerve. A SOFS does not involve the optic nerve and the vision is unaffected. With involvement of the optic nerve and a subsequent compromised vision, the condition is known as orbital apex syndrome [5].

The etiologies of the superior orbital fissure syndrome include tumours, haemorrhage, infection, trauma or idiopathic diseases [6]. In a review of 11,284 patients with craniofacial fractures, Chen and Chen found 33 cases of SOFS (0.3%) [7]. In posttraumatic SOFS, an increase in the internal orbital pressure caused by edema or bleeding at the moment of trauma may compress the nerves against the bony margin of the fissure. The extent of involvement would depend on the pressure created. Diagnosis of traumatic SOFS is based on clinical symptoms and radiographic examination. In the present case, the superior orbital fissure was intact and there were no associated fractures or haematoma shown on the CT scan. This case had no certain cause. After careful examination, we noticed that the width of the superior orbital fissure on the affected side seemed narrow. The width in the present case was 3.01 mm on the right side and 1.86 mm on the left side. For this reason, we considered that the transmitted force from the trauma and edema caused neuropraxia of the nerves in consequence of the narrow superior orbital fissure. Park and Kim demonstrated CT scans of 142 patients diagnosed with facial trauma. The widths of the superior orbital fissures were 3.78±0.92 mm on the right (maximum, 6.53; minimum, 2.04), 3.79±0.96 mm on the left side (maximum, 6.98; minimum, 2.04) and 3.79±0.93 across both sides in male and 3.69±1.18 mm on the right (maximum, 6.67; minimum, 1.95), 3.62±1.34 mm on the left (maximum, 7.47; minimum, 1.35) and 3.65±1.26 mm across both sides in female patients. They suggested that a narrow superior orbital fissure may be a risk factor for the superior orbital fissure syndrome [8].

Fujiwara et al. reported a similar case of traumatic superior orbital fissure syndrome which had no obvious cause [9]. They measured the width of the superior orbital fissure using the CT scans of 32 healthy patients and 5 cadavers. The results indicated that the width was 3.73 mm in the CT scans of healthy patients and 3.21 mm in the cadavers. The width in their patient on the affected side was 1.6 mm. They hypothesised that a narrow superior orbital fissure might reduce the tolerance to compression of the nerves by edema.

The optimal management of traumatic SOFS remains unclear and may depend on the cause. Treatment has varied from conservative treatment to steroid administration, also surgical intervention has been reported. The benefits of steroids appear to be from the antioxidant mechanism and/or the ability of such high doses to reduce edema and subsequent ischemia at the affected sites [10]. Currently, the authors administer megadose steroid with methylprednisolone (30 mg/kg intravenous loading dose followed by 15 mg/kg every 6 hours for 3 days) to help reduce the swelling that may worsen the symptoms of SOFS [11]. Surgery can be considered when there is evidence of optic nerve compromise either by bony fragments impinging on the nerve or there is an obvious retroorbital hematoma showing no signs to resolve [6]. If the patient presents with decreased visual acuity with SOFS symptoms, orbital apex syndrome should be considered. If our patient is not treated with steroid therapy, the patient might spontaneously recover from the superior orbital fissure syndrome several weeks later.

SOFS is a rare complication of craniofacial injury. Diagnosis is based on clinical presentation which can be confirmed by radiological examination. In the absence of compression by the fracture fragments and haematoma, it needs to be kept in mind that the the narrow superior orbital fissure may decrease tolerance to compression of the nerves by direct force and edema. Partial to complete recovery of the cranial nerve function can be expected after proper treatment and improvement reaches its plateau by the end of 6 months.

## Notes

### Competing interests

The authors declare that they have no competing interests.

### Informed consent

Informed consent from the patient was obtained prior to inclusion in the study and for the publication of the patient’s medical photographs.

## References

[1] Mortada A (1961). Superior orbital fissure syndrome of uncertain aetology: report of ten cases. Br J Ophthalmol.

[2] Reymond J, Kwiatkowski J, Wysocki J (2008). Clinical anatomy of the superior orbital fissure and the orbital apex. J Craniomaxillofac Surg.

[3] Morard M, Tcherekayev V, de Tribolet N (1994). The superior orbital fissure: a microanatomical study. Neurosurgery.

[4] Kurzer A, Patel MP (1979). Superior orbital fissure syndrome associated with fractures of the zygoma and orbit. Plast Reconstr Surg.

[5] Zachariades N, Vairaktaris E, Papavassiliou D, Triantafyllou K, Mezitis M (1987). Orbital apex syndrome. Int J Oral Maxillofac Surg.

[6] Rai S, Rattan V (2012). Traumatic superior orbital fissure syndrome: Review of literature and report of three cases. Natl J Maxillofac Surg.

[7] Chen CT, Wang TY, Tsay PK, Huang F, Lai JP, Chen YR (2010). Traumatic superior orbital fissure syndrome: assessment of cranial nerve recovery in 33 cases. Plast Reconstr Surg.

[8] Park Y, Kim Y (2017). A Statistical Analysis of Superior Orbital Fissure Width in Korean Adults using Computed Tomography Scans. Arch Craniofac Surg.

[9] Fujiwara T, Matsuda K, Kubo T, Tomita K, Yano K, Hosokawa K (2009). Superior orbital fissure syndrome after repair of maxillary and naso-orbito-ethmoid fractures: a case study. J Plast Reconstr Aesthet Surg.

[10] Hall ED, Braughler JM (1982). Glucocorticoid mechanisms in acute spinal cord injury: a review and therapeutic rationale. Surg Neurol.

[11] Chen CT, Chen YR (2010). Traumatic superior orbital fissure syndrome: current management. Craniomaxillofac Trauma Reconstr.

